# Use of KRD-PACE as Salvage Therapy in Aggressive, Relapsed/Bortezomib-Refractory Extramedullary Multiple Myeloma: A Report of Two Cases and Literature Review

**DOI:** 10.1155/2020/4360926

**Published:** 2020-02-18

**Authors:** Ricardo D. Parrondo, Vivek Roy, Taimur Sher, Victoria Alegria, Asher A. Chanan-Khan, Sikander Ailawadhi

**Affiliations:** ^1^Department of Hematology-Oncology, Mayo Clinic Florida, Jacksonville, FL, USA; ^2^Department of Hematology-Oncology, St. Vincent's Riverside, Jacksonville, FL, USA

## Abstract

Extramedullary multiple myeloma is defined by the presence of plasma cell infiltration outside of the bone marrow. It is associated with a poor prognosis and resistance to therapy and is often associated with high-risk cytogenetics. Aggressive relapsed and refractory extramedullary multiple myeloma is often treated with salvage infusional chemotherapy to achieve rapid disease control. Commonly used regimens include DCEP, CVAD, and VTD-PACE. While VTD-PACE contains bortezomib and thalidomide which have potent antimyeloma activity, the advent of novel agent therapy with proteasome inhibitors and immunomodulatory agents being used in the first-line setting has resulted in many patients being refractory to bortezomib by the time they are treated with VTD-PACE. Herein, we discuss two cases of aggressive relapsed, high-risk, bortezomib-refractory extramedullary multiple myeloma treated with KRD-PACE and review the available clinical data on salvage chemotherapy regimens used in relapsed refractory myeloma.

## 1. Introduction

Multiple myeloma (MM) is a plasma cell neoplasm that accounts for 1% of all cancers and approximately 10% of all hematologic malignancies [1]. MM is defined by the presence of ≥10% of clonal plasma cells in the bone marrow (or a biopsy proven extramedullary plasmacytoma) and by end-organ damage attributable to the MM such as anemia, lytic bone lesions, renal failure, and hypercalcemia [1]. Approximately, 1% to 2% of patients have extramedullary disease (EMD) upon initial diagnosis and 8% develop EMD later on in the disease course, typically after multiple relapses [2]. EMD is defined by the presence of soft-tissue plasmacytomas or plasma cell infiltration outside of the bone marrow [3]. The presence of high-risk cytogenetics (particularly del(17p) and amp [1q21]) is associated with development of EMD [4]. EMD is associated with an adverse prognosis in newly diagnosed and in relapsed MM patients and tends to be resistant to proteasome inhibitors, immunomodulatory agents, and even novel agents such as daratumumab [5–7]. As such, infusional “traditional” chemotherapy agents are used in the treatment of patients with relapsed/refractory MM (RRMM) as a means of rapid tumor debulking or as a bridge to high-dose therapy and stem cell transplant. Several such intensive infusional chemotherapy regimens are currently used.

In a population of patients with RRMM, hyperfractionated cyclophosphamide, vincristine, doxorubicin, and dexamethasone (CVAD) has been reported to produce an overall response rate (ORR) of 40% and a median overall survival (OS) of 15 months [8]. Dexamethasone with continuous-infusion cyclophosphamide, etoposide, and cisplatin (DCEP) demonstrated an ORR of 58% and a median response duration of 9 months in a population of RRMM patients [9]. In patients with RRMM, DT-PACE (thalidomide, dexamethasone, and 4-d continuous infusions of cisplatin, doxorubicin, cyclophosphamide, and etoposide) produced an ORR of 61% [10]. Bortezomib (V) is frequently administered with DT-PACE [11]. In RRMM, the proteasome inhibitor carfilzomib (K) has proven to produce superior response rates, progression-free survival (PFS), and OS compared to V [12]. Likewise, the addition of K to lenalidomide (R) and D produces superior response rates and PFS compared to RD in RRMM [13]. Furthermore, the overlapping toxicity of peripheral neuropathy by bortezomib and thalidomide makes them less desirable to combine in a regimen [14].

As a majority of patients may be heavily pretreated and refractory to V by the time they are considered for salvage infusional chemotherapy, we wanted to examine the efficacy of KRD-PACE as the salvage therapy for RRMM. Herein, we describe the efficacy and clinical course of two patients with aggressive, V-refractory, extramedullary, RRMM with high-risk cytogenetics who were treated with KRD-PACE and provide a succinct review of the literature.

## 2. Discussion

### 2.1. Patient 1

**Figure 1:**
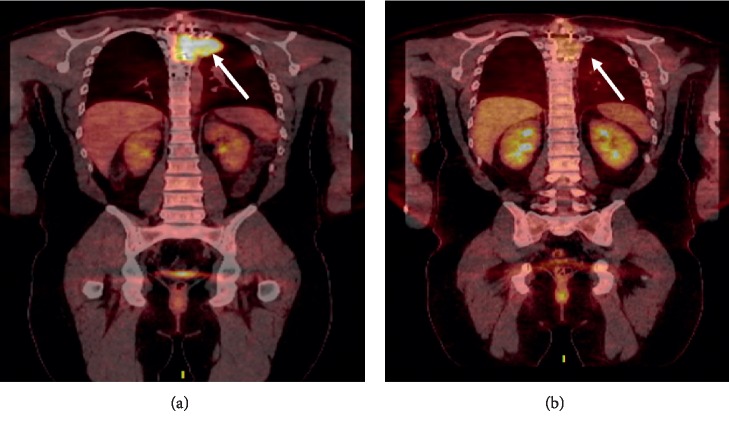
(a) 18F FDG PET-CT at relapse showing large soft-tissue mass replacing the T4 vertebral body (white arrow). (b) 18F FDG PET-CT after 2 cycles of KRD-PACE showing near-complete resolution of the extraosseous soft-tissue component involving patient's known T4 vertebral body lesion (white arrow).

A 32-year-old male was diagnosed with a solitary plasmacytoma of the left T4 paraspinal area extending into the T4 vertebral body in the July of 2016. A CT-guided biopsy of the T4 lesion was performed which revealed sheets of kappa-restricted plasma cells. A bone marrow biopsy did not reveal a clonal plasma cell population. Serum protein electrophoresis revealed an M spike of 0.7 g/dL, and immunofixation revealed an IgG kappa monoclonal protein. The patient subsequently received radiation therapy (RT) consisting of 360 cGy in 2 fractions. Prior to receiving a 3^rd^ fraction of RT, the patient developed acute onset severe back pain with bilateral lower extremity paresthesias and weakness. An MRI of the spinal canal revealed a persistent paraspinal mass and a new fracture of the T4 vertebrae. The patient subsequently underwent surgical resection of the plasmacytoma with reconstruction spinal fusion. Pathology revealed kappa-restricted plasma cells arising in sheets. The patient then underwent postoperative RT to the tumor bed receiving 3960 cGy in 22 fractions. In the May of 2018, the patient developed back pain, and an MRI of the back revealed a 5 cm × 5.7 cm mass on the left T4 hemivertebra with extra vertebral extension along the posterior left chest wall. In the June of 2018, a biopsy of this lesion revealed kappa-restricted plasma cells in sheets. Fluorescence *in situ* hybridization (FISH) of the lesion revealed monosomies 1, 8, 13, 14, and 17, which includes loss of the TP53 gene region. Restaging workup is shown in Table 1. The patient was not a candidate for additional RT or surgery to the spine, and thus was started on V-cyclophosphamide-D (CyBorD) for relapsed, extramedullary multiple myeloma. After receiving one cycle of CyBorD, the patient continued to complain of back pain and developed worsening paresthesias of the thighs bilaterally and lower extremity weakness. An MRI of the thoracic spine revealed an increase in the size of the lateral aspect of the left-sided paraspinal mass with severe cord compression from T3 through T5, and 18F FDG PET-CT images are shown in Figure 1(a). Given the patient's poor response to CyBorD and urgency of cord compression, it was decided to start the patient on salvage carfilzomib (K), lenalidomide (R), dexamethasone (D), cisplatin (P), doxorubicin (A), cyclophosphamide (C), and etoposide (E); KRD-PACE. KRD-PACE was dosed at K (20 mg/m^2^ on days 1 and 2 followed by 56 mg/m^2^ on days 8,9,15, and 16), R (25 mg daily on days 1–21), D (40 mg orally on days 1–4) with P (10 mg/m^2^/day), A (10 mg/m^2^/day), C (400 mg/m^2^/day), and E (40 mg/m^2^/day) given as a continuous intravenous infusion over 24 hours on days 1–4 (Table 2). The patient tolerated two cycles of KRD-PACE well without the need for extended hospitalization, dose reductions, transfusion, or growth factor support. He did not develop febrile neutropenia. The patient had a very good partial response (VGPR) to therapy (Table 1) with near resolution of the paraspinal plasmacytoma (Figure 1(b)). He subsequently went on to stem cell mobilization and collection followed by a melphalan 200 mg/m^2^ salvage autologous stem cell transplant (ASCT) after which he obtained a complete response. He is currently on maintenance therapy with lenalidomide 10 mg on days 1–21 and ixazomib 2 mg weekly, 13 months after KRD-PACE.

### 2.2. Patient 2

**Figure 2:**
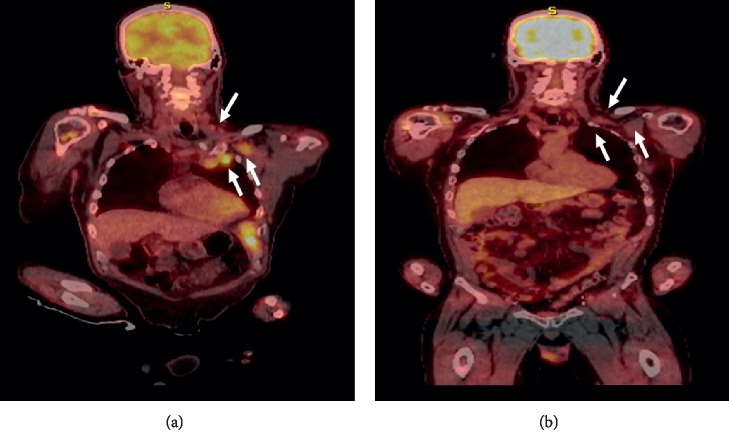
(a) 18F FDG PET-CT at relapse showing left cervical and supraclavicular adenopathy and numerous left subpectoral, axillary, and internal mammary adenopathy (white arrows). (b) 18F FDG PET-CT after 2 cycles of KRD-PACE revealing complete resolution of adenopathy.

A 56-year-old male developed temporal-occipital headaches in the April of 2017. In the November of 2017, an MRI brain was done which revealed an enlarging enhancing skull base lesion involving the clivus, right temporal bone, right petrous apex, and bilateral occipital condyles with the involvement of the right hypoglossal canal. The MRI brain also picked up bilateral upper cervical lymphadenopathy. A subsequent skeletal survey revealed multiple poorly defined lytic lesions throughout the axial and appendicular skeleton. A CT of the chest, abdomen, and pelvis revealed left axillary, right retrocrural, and left pericardial lymphadenopathy. The patient had a multiple myeloma workup (Table 1) which revealed a high-risk, revised international scoring system (R-ISS) III IgA kappa myeloma. A biopsy of one of the aforementioned cervical lymph nodes revealed sheets of kappa-restricted plasma cells, and FISH revealed a duplication 1q consistent with extramedullary disease at diagnosis. Bone marrow cytogenetics revealed a 1q duplication as well as trisomy 6, 11, and 15 and monosomy 13. The patient was subsequently started on VRD in the December of 2017 and achieved a partial response after 4 cycles. The patient had progressive disease in the April of 2018 as evidenced by a pathologic right humerus midshaft fracture. He then had surgical fixation of the fracture (pathology revealed a plasmacytoma with kappa-restricted plasma cells) and was started on daratumumab (Dara)-R-D in the May of 2018. After 4 cycles of Dara-R-D, restaging labs and an 18F FDG PET-CT revealed stable disease. After 6 cycles of Dara-R-D, the patient developed disease progression manifested by back pain for which thoracic and lumbar CT scans revealed a pathologic fracture of L5 and multiple pathologic nondisplaced fractures of the rib cage. Extramedullary disease was noted on 18F FDG PET-CT (Figure 2(a)). Restaging studies are given in Table 1. Peripheral flow cytometry did not real evidence of plasma cell leukemia. The patient was subsequently started on KRD-PACE salvage therapy with the following dosing schedule: K (20 mg/m^2^ on day 1 followed by 27 mg/m^2^ on day 2 of each cycle), R (15 mg daily on days 1–21), D (40 mg orally on days 1–4) with P (10 mg/m^2^/day), A (10 mg/m^2^/day), C (400 mg/m^2^/day), and E(40 mg/m^2^/day) given as a continuous intravenous infusion over 24 hours on days 1–4 (Table 2). He ultimately received 2 cycles of KRD-PACE. The patient developed grade 3 mucositis and anemia, grade 4 thrombocytopenia and neutropenia requiring transfusion (a total of 8 units of packed red cells and 10 units of platelets), and growth factor support (three 6 mg filgrastim injections), as well as a right upper extremity deep venous thrombosis (Table 1). He did not develop neutropenic fever. The patient was only hospitalized for 4 days during each cycle of KRD-PACE and was never rehospitalized. Transfusional and growth factor support was managed in the outpatient setting. The patient had a complete response (CR) to therapy (Table 1) with a resolution of lymphadenopathy (Figure 2(b)). He went on to stem cell mobilization but was unable to collect an adequate number of stem cells on two occasions. He remained in CR of therapy but developed an extramedullary relapse 6 months after completion of KRD-PACE and is now being considered for enrollment in an anti-B-cell maturation antigen (BCMA) chimeric antigen receptor T-cell (CAR-T) therapy trial.

## 3. Conclusion

We describe two cases of aggressive, relapsed, bortezomib-refractory extramedullary MM with high-risk cytogenetics who achieved deep responses with KRD-PACE salvage therapy. One of the patients, a 32-year-old male with minimal MM bone marrow involvement and less prior treatment, tolerated 2 cycles of KRD-PACE without any complications or need for transfusional support. He achieved a VGPR and proceeded to high-dose therapy and ASCT to which he achieved a CR. The 56-year-old patient had extensive bone marrow involvement and 2 prior lines of treatment. He tolerated 2 cycles of KRD-PACE without the need for extended hospitalization, but developed profound cytopenias which required 34–48 days to recover and multiple red cell and platelet transfusions as well as granulocyte colony stimulating factor support. He achieved a complete response which lasted for 6 months without additional therapy.

DCEP, CVAD, and VTD-PACE-like regimens are the most commonly used infusional salvage chemotherapy regimens in patients with RRMM. In a retrospective analysis of 107 patients with RRMM, the ORR, PFS, and OS were 52%, 3.8 months, and 8.9 months for DCEP; 49%, 5.8 months, and 8.3 months for CVAD; and 73%, 4.5 months, and 8.5 months for VTD-PACE, respectively [15]. There were no significant differences in response rates or survival benefit amongst the three regimens [15]. Patients received a median number of 2 cycles, >70% were refractory to V and >60% were refractory to R [15]. Successful autologous transplant after salvage chemotherapy was associated with superior PFS (HR, 0.25; 95% CI, 0.14–0.45) and OS (HR 0.19, 95% CI, 0.12–0.30) [15]. High-risk cytogenetics (*P*=0.05) and EMD (*P*=0.006) were the factors associated with inferior OS in the study population [14]. Common adverse effects were increased transfusion requirements (62%), febrile neutropenia (37%), delays in treatment (36%), rehospitalization (34%), venous thromboembolism (6%), dose reductions due to adverse events (5%), and treatment-related mortality (7%) [15]. There was a trend toward more febrile neutropenia with VTD-PACE and prolonged hospitalization among patients treated with CVAD, but there were no statistically significant differences in adverse events among the 3 regimens [15]. In a retrospective study of 141 patients with RRMM treated with VTD-PACE-like regimens, ORR was 54%, OS was 8.1 months, and PFS was 3.1 months [16]. Most patients received 1–2 cycles of a VTD-PACE-like regimen, 85% were refractory to V, and 60% were refractory to R. Patients required a median of 4 packed red cell transfusions and 4 platelet transfusions during cycle 1 [16]. Median time to recovery of absolute neutrophil count and platelet count from initiation of a VTD-PACE-like regimen was 18 (range, 12–44) and 21(range 11–45) days, respectively [16]. Age ≥60 (HR 2.28, 95% CI, 1.41–3.70) and R–ISS III (HR 2.38, 95% CI, 1.35–4.04) predicted shortened OS [16]. Presence of EMD and high-risk cytogenetics, and being refractory to proteasome inhibitors or immunomodulatory agents were not associated with shortened OS [16].

Salvage chemotherapy regimens, including the most intensive regimen, VTD-PACE, confer a median OS of 8 months and a median PFS of 3–4 months for RRMM patients based on the current published studies [15,16]. OS and PFS are even shorter for patients with high-risk cytogenetics and EMD. Given that over 60% of patients in the aforementioned studies were refractory to V and R when they received salvage regimens, substitution of VT for KR to make KRD-PACE is a plausible alternative. A study by Cowan et al. examined the use of KRD-PACE for chemomobilization in 20 patients with RRMM [17]. 15 patients received 1 cycle of KRD-PACE, and 5 patients received 2 cycles. The ORR was 25% with 75% achieving stable disease. 100% of patients underwent ASCT. No patients experienced cardiac toxicity. Following transplant, the median time to neutrophil engraftment was 18 days and the median time to platelet engraftment was 13 days. The PFS at 6 months was 63% (95% CI, 0.382–1), and the OS at 6 months was 91% (95% CI, 0.754–1) [17]. Harrell et al. reported a retrospective study of 52 patients with RRMM who were treated with KD-PACE-like regimens [18]. In this study, 54% of patients had high-risk cytogenetics and had received a median of 3 prior lines of therapy and 64% had KD-PACE together with an immunomodulatory agent. Grade 3/4 toxicities included neutropenia (93%), thrombocytopenia (87%), and anemia (37%). Febrile neutropenia occurred in 35%. The overall response rate (≥PR) was 77% including CR (12%), VGPR (23%), and PR (42%). Median PFS was 4.6 mo (95% CI 3.2–7.5 mo) and median OS 11.2 mo (95% CI 6.1–14.5 mo) [18].

Relapsed refractory extramedullary MM with high-risk cytogenetics is a highly aggressive, treatment-refractory disease state with a dismal prognosis. Salvage chemotherapy followed by ASCT is one of the few treatment options that can prolong survival and act as a bridge to a clinical trial. While most patients are refractory to V by the time they are treated with salvage regimens, KRD-PACE offers an efficacious option for treatment that introduces a novel agent (K) highly active in RRMM. In the ASPIRE and ENDEAVOR trials, K has proven to produce superior ORR, OS, and PFS compared to V-based or R-based regimens in RRMM [12,13]. Furthermore, in the ASPIRE trial, KRD was superior to RD for PFS across high-risk cytogenetic risk groups, suggesting that this combination partly abrogates the negative impact of t(4; 14) and del(17p) [13]. Prospective trials evaluating the safety and efficacy of salvage KRD-PACE in RRMM, especially patients with high-risk myeloma and those with EMD, are warranted.

## Figures and Tables

**Table 1:** Response measurement and hematologic toxicity/recovery time after 2 cycles of KRD-PACE.

Patient 1			Patient 2		
Parameter	Staging studies at relapse before KRD-PACE	Staging studies after 2 cycles of KRD-PACE	Parameter	Staging studies at relapse before KRD-PACE	Staging studies after 2 cycles of KRD-PACE
ISS score (at diagnosis)	I	—	ISS score (at diagnosis)	III	—
Revised-ISS score (at diagnosis)	II	—	Revised-ISS Score (st diagnosis)	III	—
Cytogenetics/FISH (at diagnosis)	Monosomies 1, 8, 13, 14 and 17, which includes loss of the TP53 gene region	—	Cytogenetics/FISH (at diagnosis)	1q duplication. In addition, trisomy 6, 11, and 15, monosomy 13	—
Hgb	14.2 g/dL	11.1 g/dL	Hgb	13.0 g/dL	11.1 g/dL
Plt	172 × 10^9^/L	312	Plt	169 × 10^9^/L	68 × 10^9^/L
Cr	0.86 mg/dL	0.74 mg/dL	Cr	0.96 mg/dL	1.06 mg/dL
LDH	635 U/L	184 U/L	LDH	235 U/L	159 U/L
B_2_-microglobulin	1.55 mcg/mL	1.27 mcg/mL	B_2_-microglobulin	9.41 mcg/mL	2.37 mcg/mL
M-spike	0.9 g/dL	0.1 g/dL	M-spike	0.2 g/dL	0.1 g/dL
IFE	IgG kappa	IgG kappa	IFE	IgA kappa	No monoclonal protein
IgG	1410 mg/dL	544 mg/dL	IgG	257 mg/dL	294 mg/dL
IgA	155 mg/dL	92 mg/dL	IgA	584 mg/dL	9 mg/dL
IgM	80 md/dL	24 mg/dL	IgM	24 md/dL	9 mg/dL
Lambda	0.738 mg/dL	0.283 mg/dL	Lambda	0.248 mg/dL	0.042 mg/dL
Kappa	22.9 mg/dL	0.701 mg/dL	Kappa	1.25 mg/dL	<0.030 mg/dL
K/L ratio	31.02	2.48	K/L Ratio	5.04	<0.712
Bone marrow biopsy	2-3% kappa-restricted atypical plasma cells.	—	Bone marrow biopsy	—	No atypical plasma cells detected.
Response	—	Very good partial response	Response	—	Complete response
Plt nadir/time to recovery	—	69 × 10^9^/L/7 days	Plt nadir/time to recovery	—	8 × 10^9^/L/45 days
ANC nadir/time to recovery	—	1.18 × 10^9^/L	ANC nadir/time to recovery	—	0.04 × 10^9^/34 days
Hgb nadir/time to recovery	—	11.2 g/dL	Hgb nadir/time to recovery	—	6.3 g/dl/48 days

**Table 2:** KRD-PACE dosing schedule for patient 1 and KRD-PACE dosing schedule for patient 2.

28d cycle	K	R	D	P	A	C	E
*A: Patient 1, KRD-PACE schedule*							
Dose	20 mg/m^2^ (day 1) then 56 mg/m^2^ thereafter	25 mg	40 mg	10 mg/m^2^	10 mg/m^2^	400 mg/m^2^	40 mg/m^2^
Schedule	Days 1, 2, 8, 9, 15, 16	Days 1–21	Days 1–4	Days 1–4	Days 1–4	Days 1–4	Days 1–4

*B: Patient 2, KRD-PACE schedule*							
Dose	20 mg/m^2^ (day 1) then 27 mg/m^2^	15 mg	40 mg	10 mg/m^2^	10 mg/m^2^	400 mg/m^2^	40 mg/m^2^
Schedule	Days 1,2	Days 1–21	Days 1–4	Days 1–4	Days 1–4	Days 1–4	Days 1–4
